# ROR1 is Expressed in Diffuse Large B-Cell Lymphoma (DLBCL) and a Small Molecule Inhibitor of ROR1 (KAN0441571C) Induced Apoptosis of Lymphoma Cells

**DOI:** 10.3390/biomedicines8060170

**Published:** 2020-06-23

**Authors:** Amineh Ghaderi, Amir Hossein Daneshmanesh, Ali Moshfegh, Parviz Kokhaei, Jan Vågberg, Johan Schultz, Thomas Olin, Sara Harrysson, Karin E Smedby, Elias Drakos, Georgios Z. Rassidakis, Anders Österborg, Håkan Mellstedt, Mohammad Hojjat-Farsangi

**Affiliations:** 1 Department of Oncology-Pathology, BioClinicum, Karolinska University Hospital Solna and Karolinska Institutet, 17164 Stockholm, Sweden; amineh.ghaderi@ki.se (A.G.); prince_ahdm@yahoo.com (A.H.D.); ali.moshfegh@ki.se (A.M.); parviz.kokhaei@ki.se (P.K.); hdrakos@med.uoc.gr (E.D.); georgios.rassidakis@ki.se (G.Z.R.); anders.osterborg@sll.se (A.Ö.); mohammad.hojat-farsangi@ki.se (M.H.-F.); 2 Kancera AB, Karolinska Institute Science Park, 171 48 Solna, Sweden; jan.vagberg@kancera.com (J.V.); johan.schultz@kancera.com (J.S.); thomas.olin@kancera.com (T.O.); 3 Department of Immunology, Semnan University of Medical Sciences, Semnan 35147-99442, Iran; 4 Division of Clinical Epidemiology, Department of Medicine Solna, Karolinska Institutet, 171 76 Stockholm, Sweden; sara.harrysson@sll.se (S.H.); karin.ekstrom.smedby@ki.se (K.E.S.); 5 Department of Hematology, Karolinska University Hospital Solna, 171 77 Stockholm, Sweden; 6 Department of Pathology, Medical School, University of Crete, 71110 Heraklion Crete, Greece

**Keywords:** DLBCL, ROR1, small molecules

## Abstract

The receptor tyrosine kinase ROR1 is absent in most normal adult tissues, but overexpressed in several malignancies. In this study, we explored clinical and functional inhibitory aspects of ROR1 in diffuse large B-cell lymphoma (DLBCL). ROR1 expression in tumor cells was more often observed in primary refractory DLBCL, Richter’s syndrome and transformed follicular lymphoma than in relapsed and non-relapsed DLBCL patients (*p* < 0.001). A survival effect of ROR1 expression was preliminarily observed in relapsed/refractory patients independent of gender and stage but not of age, cell of origin and international prognostic index. A second generation small molecule ROR1 inhibitor (KAN0441571C) induced apoptosis of ROR1+ DLBCL cell lines, similar to venetoclax (BCL-2 inhibitor) but superior to ibrutinib (BTK inhibitor). The combination of KAN0441571C and venetoclax at EC_50_ concentrations induced almost complete killing of DLBCL cell lines. Apoptosis was accompanied by the downregulation of BCL-2 and MCL-1 and confirmed by the cleavage of PARP and caspases 3, 8, 9. PI3Kδ/AKT/mTOR (non-canonical Wnt pathway) as well as β-catenin and CK1δ (canonical pathway) were inactivated. In zebra fishes transplanted with a ROR1+ DLBCL cell line, KAN0441571C induced a significant tumor reduction. New drugs with mechanisms of action other than those available for DLBCL are warranted. ROR1 inhibitors might represent a novel promising approach.

## 1. Introduction

Receptor tyrosine kinases (RTKs) are important structures for targeted cancer therapy. Both monoclonal antibodies and small molecules have been clinically successful against several RTKs in a variety of tumors [1].

ROR1 and ROR2 belong to the receptor tyrosine kinase-like orphan receptor (ROR) family of RTK which were identified based on homology to the neurotrophin receptors (NTRK), and are essential for the development of the nervous system. ROR proteins are important for cell polarity, division, and migration of neurons [2]. The ROR family acts as receptors for Wnt soluble proteins [3]. The RTK ROR1 is of importance during embryogenesis but is downregulated in most adult cells [4]. A recent study described the expression of ROR1 in several normal tissues as pancreatic islets, parathyroid glands and regions of the esophagus, stomach and duodenum by immunohistochemistry using an antibody targeting the C-terminal of ROR1 [5]. However, ROR1 is uniquely expressed in various tumor cells compared to normal post-partum tissues [6,7,8,9,10] and is shown to be of significance for tumor cell survival and proliferation [11,12,13] as well as epithelial-to-mesenchymal transition (EMT), migration and metastasis [14]. ROR1 is also involved in maintaining the stemness of cancer cells, and is shown to be associated with disease progression and chemoresistance of various tumor cells [15,16,17]. Both Wnt canonical, RhoA/Rac1 GTPases, and PI3K/AKT/mTOR signaling pathways and activation of CREB, C-Jun transcription factors as well as transcriptional coactivator YAP/TAZ have been suggested to be associated with ROR1 activation [7,18,19,20,21,22].

ROR1 is expressed in B cell malignancies including chronic lymphocytic leukemia/small lymphocytic lymphoma (CLL/SLL), mantle cell lymphoma (MCL), acute lymphoblastic leukemia (ALL) and recently also diffuse large B cell lymphoma (DLBCL) as well as in solid tumors such as lung, pancreatic, ovarian, and breast cancers [6,7,10,11,18,19,23]. siRNA transfection of tumor cells of both hematological and epithelial origin induced significant tumor cell death [10,18,24,25]. In several tumors, high expression of ROR1 correlated with short survival [10,25] or disease progression [26].

A humanized monoclonal antibody against ROR1 (cirmtuzumab) inhibited RhoA and hematopoietic-lineage-specific Lyn substrate-1 (HS1) as well as inhibition of CLL stemness gene signatures [15]. HS1 has been shown to be associated with an adverse prognosis of CLL patients [27,28]. We recently described a first-in-class ROR1 small molecule inhibitor (SMI) (KAN0493834) inducing specific apoptosis of CLL cells and tumor cell reduction in immunodeficient mice xenotransplanted with human CLL cells [29]. Our ROR1 SMI shows binding to ROR1, dephosphorylation of ROR1 [29] and in silico modeling high fitting of the SMI to the ATP pocket of the TK domain [29].

DLBCL is the most common type of non-Hodgkin lymphoma (NHL). In patients with DLBCL, there is a great medical need to develop new treatment alternatives for those not responding to or relapsing after first-line immunochemotherapy [30]. In the present study, ROR1 expression was evaluated in tumor material from DLBCL patients with different clinical presentation and outcome. The anti-tumor effects of a second generation of ROR1 SMI, KAN0441571C (Table S1), was tested in in vitro and in vivo preclinical DLBCL models for the induction of tumor cell apoptosis as well as in combination with targeted therapeutics (BTK and BCL-2 inhibitors) with mechanisms of action other than ROR1 inhibitors. Our results indicate that the second generation ROR1 inhibitor might be a promising new drug candidate that warrants further evaluation in clinical trials in high-risk DLBCL patients.

## 2. Materials and Methods

### 2.1. Patients

The diagnosis of DLBCL was based on the updated World Health Organization (WHO) classification [31]. Patients were diagnosed and treated at the Department of Hematology, Karolinska University Hospital Solna, Stockholm, Sweden, according to Swedish national guidelines (ethic committee: Etikprovnings myndigheten, ethic number: 00-138, 2016-12-20), during the period 2007–2014. Three groups of de novo DLBCL patients receiving primary treatment with curative intent were retrospectively identified in the regional lymphoma register based on available medical records and archived tissue samples. In total, 63 de novo (non-transformed) DLBCL patients were enrolled: 30 patients did not experience treatment refractoriness or relapse after a median follow-up time of 32 months (range 21–49). Eleven patients were primary refractory to first-line therapy and 22 patients experienced relapse during a follow-up period of five years. Retrospectively, IHC staining for ROR1 was performed on tumor tissue from the primary diagnosis. To investigate the full spectrum of DLBCL including transformation, we added 10 patients with Richter’s transformation (RT) of CLL/SLL and 11 with aggressive transformation of pre-existing low-grade follicular lymphoma (FL). Samples from the last two diagnostic groups were obtained at the time of diagnosis of transformation. A hypothesis-generating approach was applied to study the relationship between ROR1 expression at diagnosis and tumor aggressiveness. The two poor-prognosis de novo DLBCL subgroups [relapsed/refractory(R/R)] were merged for a retrospective analysis of outcome in relation to ROR1 expression and other prognostic markers. Non-relapsed patients were not included for power reasons (a large prospective study would be required) in this exploratory outcome analysis and transformed DLBCL were also excluded as a heterogeneous diagnostic subgroup.

### 2.2. Tissue Microarray (TMA) and Immunohistochemical (IHC) Assays

DLBCL tissue samples taken prior to start of first-line therapy were identified and analyzed using full tissue sections or tissue microarrays (TMA) and immunohistochemistry after antigen retrieval as previously described [32]. Reactive lymph nodes (*n* = 2) and tonsils (*n* = 2) were included as controls. The use of the samples was in accordance with the Declaration of Helsinki and approved by the national ethics committee (www.etikprovningsmyndigheten.se). ROR1 expression was assessed by IHC using a polyclonal antibody against ROR1 (Proteintech, Manchester, United Kingdom). Positivity was defined as any level of unequivocal cytoplasmic and/or membranous staining in the neoplastic B cells. A 10% cutoff was used to define positivity.

### 2.3. Cell Lines

Five DLBCL cell lines obtained from ATCC were used for in vitro analyses. SUDHL4 (GCB type) ROR1^+^; MS (GCB type) ROR1^+^; RC-K8 (GCB type) ROR1^+^; OCI-LY3 (ABC type) ROR1^+^; U2932 (ABC type) ROR1^−^. ROR1 expression was analyzed by flowcytometry and Western blot (see below) including expression of phosphorylated ROR1 protein (pROR1) [29]. Cells were cultured in RPMI-1640 medium (Life Technologies, Karlsruhe, Germany), supplemented with 10% fetal calf serum (Life Technologies), penicillin (100 IU/mL) and streptomycin (100 µg/mL) (Life Technologies).

### 2.4. Small Molecule ROR1 Tyrosine Kinase Inhibitors (KAN0439834 and KAN0441571C)

The development of the first small molecule inhibitor of the tyrosine kinase ROR1 (KAN0439834) was recently described [29]. Following a high-throughput screening campaign against the tyrosine kinase domain of ROR1, more than 2000 compounds were synthesized in the hit-to-lead and lead optimization stages. Since the discovery of KAN0439834, approximately 950 additional compounds have been produced and tested for cytotoxic effect against primary cells from patients as well as peripheral blood mononuclear (PBMC) from healthy donors. The chemistry development led to the discovery of the second-generation ROR1 inhibitor, KAN0441571C. The improved second generation of ROR1 inhibitors (KAN0441571C) showed higher cytotoxic potency against various cancer cells in vitro as Hodgkin lymphoma [33], CLL, pancreatic carcinoma, and lung cancer cells as well as a substantially longer halftime (T1/2) in the mouse (11 h compared to 2.1 h) (data not shown), compared to the first generation of ROR1 inhibitor (KAN0439834). Physicochemical differences between the two compounds are summarized in Table S1. The kinase selectivity profile (specificity) of KAN0441571C is similar to the first generation ROR1 inhibitor KAN0439834 [29] (see Table S3 for details). Using five different DLBCL cell lines, KAN0441571C had a superior or similar cytotoxic potency compared to KAN0439834 (Figure S1). KAN0441571C was used in the present study for in vitro and in vivo experiments.

### 2.5. Cell Surface Markers (Flow Cytometry)

ROR1 surface staining was carried out as described previously [29]. Briefly, 10^6^ cells were washed and suspended in 100 µL of phosphate-buffered saline (PBS). Allophycocyanin (APC) conjugated anti-ROR1 (Miltenyi Biotec, Bergisch Gladbach, Germany), PE/Cy7 conjugated anti-CD19, were added and incubated for 20 min at room temperature (RT). The cells were then washed with fluorescence-activated cell sorting (FACS) buffer and counted in a FACS Canto II flow cytometry (BD Biosciences, San Jose, CA, USA). The FlowJo software program (Tree Star Inc., Ashland, OR, USA) was used for analysis of cells.

### 2.6. SDS-PAGE and Western Blot

Western blot experiments were performed as previously described [29]. DLBCL cell lines were lysed on ice for 30 min in buffer containing 1% Triton X-100, 150 mM NaCl, 50 mM Tris-HCl, 5 mM EDTA, 1% protease inhibitor cocktail (Sigma-Aldrich, St. Louis, USA), and phosphatase inhibitors (Roche Ltd., Basel, Switzerland) and centrifuged at 13000 rpm. Supernatants were collected and protein concentration measured by the BCA Protein Assay Kit (ThermoFisher Scientific, IL, USA). Twenty µg of the lysate were loaded onto 8–10% BisTris SDS-PAGE gel (ThermoFisher Scientific) and run at 160 V and 160 mA for 2 h. Electrophoresed proteins were transferred to PVDF membranes (Millipore Corporation, MA, USA) and blotted at 45 V and 145 mA for 1.5 h in Transblot cell (ThermoFisher Scientific) at RT. Membranes were blocked in blocking buffer (5% bovine serum albumin (BSA) (Santa Cruz Biotechnology, CA, USA) in PBS or tris-buffered saline (TBS) with 0.1% Tween 20 (PBS-T, TBS-T) at RT for 2 h. Membranes were probed with the respective primary antibodies overnight at 4 °C and washed five times for 1 h in PBS-T or TBS-T at RT and incubated with secondary antibody conjugated with peroxidase (Dako Cytomation, Glostrup, Denmark) for 1 h in blocking buffer at RT. Membranes were washed as described before and developed using the ECL chemiluminescence detection system (GE Healthcare, Uppsala, Sweden). The following antibodies were used; ROR1 (R&D Systems, Minneapolis, MN, USA), phospho (p) ROR1 (Tyr 641, 646 and Ser652) [29], myeloid cell leukemia (MCL)-1, cleaved poly ADP ribose polymerase (PARP), B-cell lymphoma (BCL)-2, BCL-xL, cleaved caspases 3, 8, 9, BAX, LDL receptor related protein (LRP) 6, pLRP6 (Ser 1490), SRC, pSRC (Tyr 416), Phosphoinositide 3-kinase (PI3K) p110δ, protein Kinase C (PKC)δ, pPKCδ (Tyr 311), AKT, pAKT (Ser 473), mammalian target of rapamycin (mTOR), p-mTOR (Ser 2448), cAMP response element-binding protein (CREB), pCREB (Ser 133), casein kinase (CK)1δ, β-catenin (Cell Signaling Technology, MA, USA), and pPI3Kp110δ (Tyr 485) (Santa Cruz). For loading control, membranes were probed for β-actin (Sigma-Aldrich). Densitometric qualification was carried out using the Image J1.44p software (National Institute of Health, USA). Ratios were calculated between phosphorylated and total protein.

### 2.7. MTT Cytotoxicity Assay

The MTT [3-(4,5-Dimethylthiazol-2-yl)-2,5-Diphenyltetrazolium Bromide] assay (Sigma-Aldrich) was used to measure cytotoxic capability of the ROR1 tyrosine kinase inhibitors (TKI) including KAN0439834 and KAN0441571C. Briefly, 2 × 10^4^ DLBCL cells were incubated in triplicates in 200 µL of RPMI-1640 containing 10% FCS in 96 well plates. ROR1-TKI (dissolved in dimethyl sulfoxide/DMSO) were added to the cells. Cells treated with DMSO were used as negative control. Cells were incubated for 24, 48 and 72 h. An amount of 20 µL MTT solution was added and the cell suspension incubated for 4 h at 37 °C. The reaction was stopped by adding 100 µL MTT solvent (10% SDS in 0.01 M HCL) and incubated for further 2–4 h. The plate was then read by a microplate reader at 570 nm.

### 2.8. Apoptosis Assay (Flow Cytometry)

An amount of 1 ml (1 × 10^5^ cells/mL) of DLBCL cells were seeded in each well of 6-well plates with and without compounds for 24 h. Cells were collected, washed with PBS, suspended in 100 µL of Annexin-V binding buffer (BD Biosciences) containing FITC-conjugated Annexin-V and PI (BD Biosciences) and incubated at room temperature in the dark for 20 min. After 20 min of incubation, 150 µL Annexin-V binding buffer was added. Viable cells were identified as the double negative Annexin-V and PI population. Samples were analyzed by a FACS Canto II Flow-cytometer (BD Biosciences).

### 2.9. Apoptosis of DLBCL Cells Co-Cultured with Stromal Cells

The human stromal cell line HS-5 (ROR1 negative) and OCI-Ly3 DLBCL cell line were treated with KAN0441571C (25–250 nM) in RPMI-1640 containing 10% FBS, seeded into 24-wells plate at a ratio of 1:5 and incubated at 37 °C in 5% CO2 for 24 h. Cells were harvested and stained for Annexin V/PI in ROR1^+^/CD19^+^ gated cells. Untreated and treated HS-5 as well as OCI-Ly3 cells alone, respectively served as controls.

### 2.10. Proximity Ligation Assay (PLA)

A PLA test was applied to analyze approximation of proteins in a cell or on the cell surface. OCI-Ly3 cells were cultured on sterile 8 wells glass slide (BD Biosciences) for 24 h to form a monolayer. Cells were then treated with 100–500 nM KAN0441571C for 6 h. Untreated cells were used as negative control. Slides were fixed with 4% formaldehyde, washed 3 times with PBS and blocked with blocking buffer (2% BSA, 0.1 & Tween 20 and 0.01% azide) for 1 h at RT. Cells were then incubated overnight at 4 °C with a mixture of ROR1 and LRP6 primary antibodies (1:100) (Sigma-Aldrich). PLA was performed using the Duolink^®^ In Situ Red Starter Kit Mouse/Rabbit (Sigma-Aldrich) according to the manufacturer´s protocol. After overnight incubation with the primary antibodies, slides were washed and PLA probes added and incubated at 37 °C for 1 h. Ligation reaction was carried out for 30 min after washing and the amplification process was run for 100 min. Cells were washed and nuclei was counterstained with VectaShield H-1000 mounting media containing DAPI (Vector Laboratories, Burlingame, CA, USA).

### 2.11. Zebrafish Transplantable Tumor Growth Model

First zebra fish embryos were injected at 48 h post fertilization with approximately 500 stained OCI-LY3 tumor cells in 5 nL medium in the egg yolk to validate if the cell line could form tumors. The tumors were photographed and embryos transferred to individual wells in a 24-well plate in 500 µL of PTU-treated, E3 medium (Sigma-Aldrich). Tumors were visualized again at 24 and 72 h post implantation by fluorescence microscopy. Tumor cells were analyzed with a software designed to assess tumor size in zebra fish embryos (Bioreperia, Linköping, Sweden). Tumor growth was determined by normalizing the area of the tumors at each given time point to that of the same tumor at day 0. Tumor cells grew well in the yolk and the volume increased by 70% between day 0 and day 3.

In the next step, zebra fish embryos (*n* = 4 × 21) were injected with tumor cells as above. KAN0441571C was added to the medium at 4 different concentrations: 25, 100, 250, 1000 nM. One group (*n* = 21) was untreated. Tumors were visualized by fluorescence microscopy (photographed) at day 0 and at 24 and 72 h post implantation. Tumor size was determined as above.

### 2.12. Statistical Analysis

*Chi*-Square test was applied for comparison of ROR1 expression in various clinical patient subgroups. The non-parametrical Mann–Whitney test was used for the statistical associations of variables among different groups, using R version 3.3.2 (The R Foundation for Statistical Computing, Vienna). Student’s *t*-test and Mann–Whitney *U* test were used for comparison of EC_50_ values (GraphPad Software, Inc., La Jolla, CA, USA). Overall survival (OS) was analyzed in R/R DLBCL patients only to ensure a poor-prognosis patient cohort with sufficient numbers of events in the ROR1^+^ and ROR1- groups, respectively. Survival was measured from the time of occurrence of relapsed/refractory DLBCL until death or last follow-up and was illustrated graphically by the Kaplan–Meier method. Statistical significance was estimated by the log-rank test. Cox regression models were applied for multivariable analyses. *p*-values ˂ 0.05 were considered significant. Bonferroni method was used to correct for multiple comparisons.

## 3. Results

### 3.1. Expression of ROR1 in Relation to Clinical Subgroups and Prognosis

**Figure 1:**
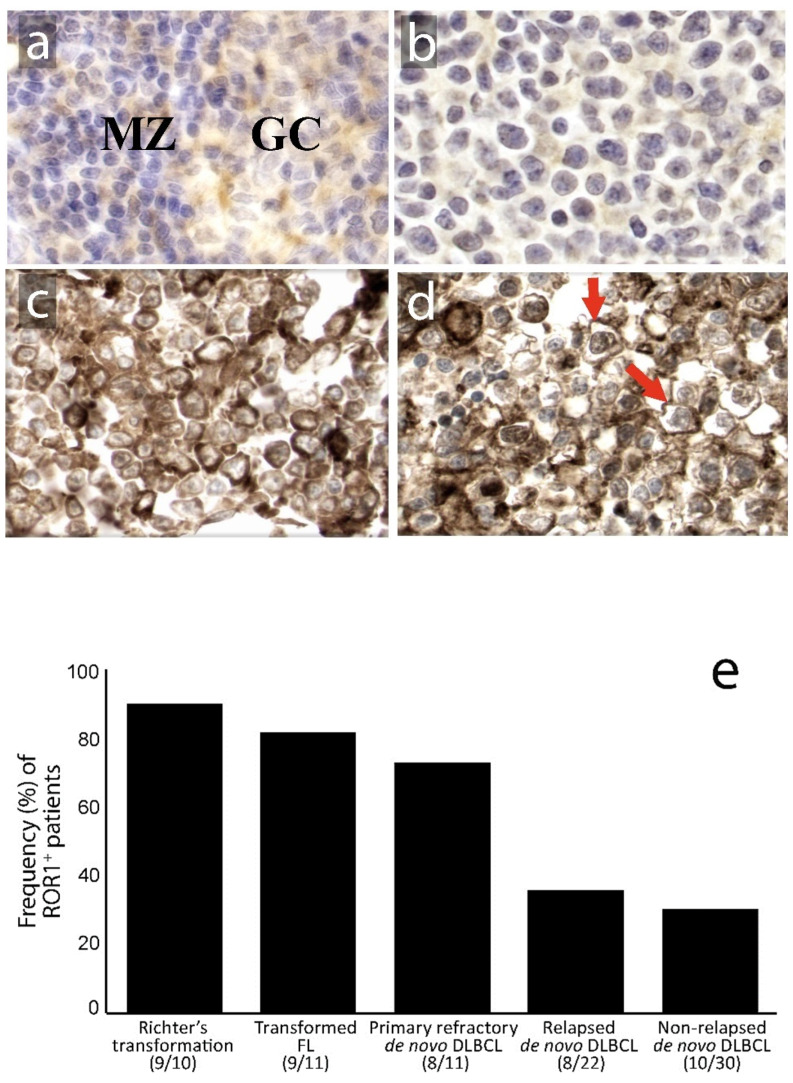
Expression (Immunohistochemical (IHC)) of the ROR1 protein in reactive lymphoid tissue and diffuse large B-cell lymphoma (DLBCL) lymphoma tissues. (magnification × 400; 3,3’-Diaminobenzidine (DAB) as chromogen, hematoxylin as counterstain). ROR1 was expressed (**a**) weakly in the cytoplasm of a subset of centroblasts in the germinal center (GC) and rare small lymphocytes in the mantle zone (MZ) of reactive lymph nodes; (**b**) representative case of DLBCL negative for ROR1; (**c**) DLBCL positive for ROR1 with predominantly cytoplasmic staining and (**d**) with a predominantly membranous staining pattern (red arrows); (**e**) ROR1 expression in tumor cells was more often observed in primary refractory DLBCL, Richter’s syndrome and transformed follicular lymphoma than in relapsed and non-relapsed DLBCL patients (*p* < 0.001, *Chi*-Square test). Numbers in brackets represent the number of ROR1-positive cases compared to the total number of cases in each group.

ROR1 was detected only faintly in the germinal center of reactive follicles of non-malignant lymphoid tissues, mostly with a cytoplasmic staining pattern (Figure 1a). In the lymphoma tissues, there were ROR1-negative cases (Figure 1b) as well as those with both cytoplasmic and membranous staining patterns for ROR1 (Figure 1c,d). ROR1 expression was more frequently observed in primary refractory DLBCL (7/11, 83%), Richter´s syndrome (9/10, 90%) and transformed follicular lymphoma (9/11, 82%) than in relapsed (8/22, 36%) and non-relapsed DLBCL patients (10/30, 33%) (*p* < 0.001) (Figure 1e). Thus, in primary refractory de novo DLBCL patients, a significantly higher frequency of ROR1^+^ patients was seen compared to non-refractory and relapsed patients (*p* < 0.05). No significant association between ROR1 expression and cell of origin (germinal center vs. non-germinal center) phenotype was noted in the non-relapsed and relapsed/primary refractory subgroups.

We next analyzed the relationship between ROR1 expression and overall survival. This analysis, which shall be regarded as strictly hypothesis-generating only due to the limited number of patients and its retrospective design, was performed in R/R de novo DLBCL patients to obtain a minimum number of outcome events. Transformed DLBCL (Richter’s syndrome, transformed FL) was not included as a prognostically heterogenous subset. The five-year OS rate was 42% for patients with ROR1^−^ DLBCL (*n* = 17) as compared to 7% for those with ROR1^+^ tumors (*n* = 16) (*p* < 0.05, log-rank test) (data not shown). In a multivariable analysis, ROR1 positivity was independent of gender and Ann Arbor stage but dependent on age and International Prognostic Index (IPI) (Table S2).

### 3.2. Cytotoxicity of KAN0441571C in DLBCL Cell Lines

**Figure 2:**
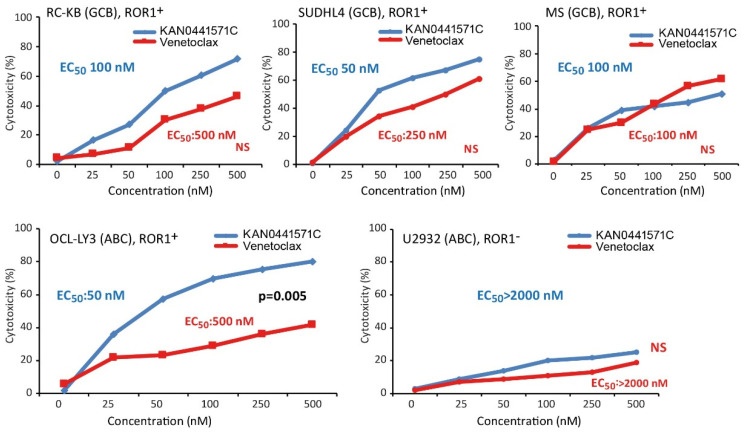
Cytotoxicity (MTT) of DLBCL cell lines of the GCB and ABC types incubated with KAN0441571C and venetoclax respectively for 48 h. EC_50_ values for the respective drug are indicated as well as significant levels comparing KAN0441571C and venetoclax. All cell lines express phosphorylated ROR1 with the exception for U2932. For each concentration, six replicates were used and each experiment was carried out twice. NS: Not significant.

**Figure 3:**
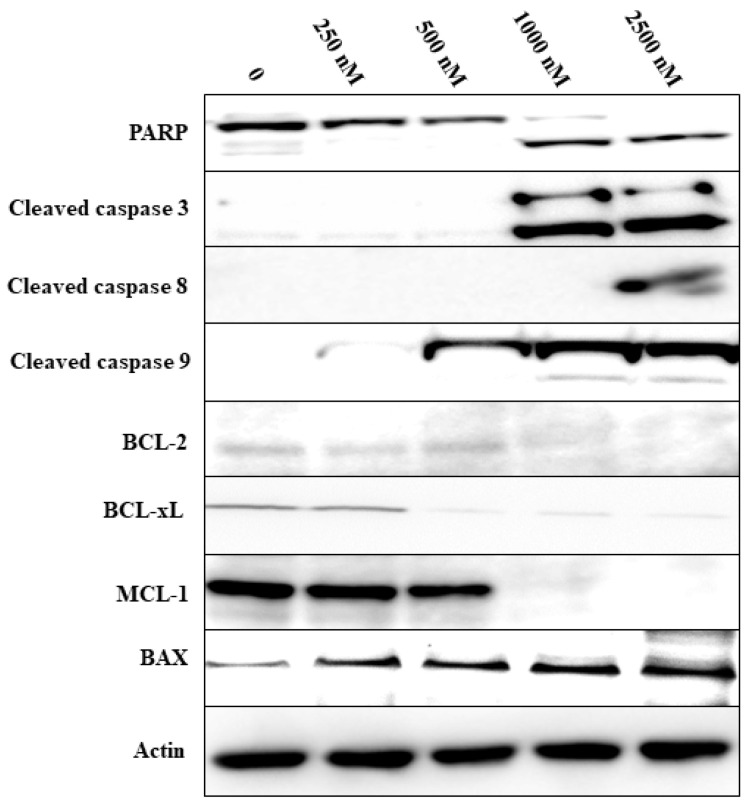
Effects of KAN0441571C on apoptotic associated proteins mantle cell lymphoma (MCL)-1, BCL-2, BCL-xL, caspases-3, 8,9 and cleaved poly ADP ribose polymerase (PARP) after 24 h of incubation (OCI-LY3 cell line). MCL-1, BCL-2 and BCL-xL proteins were downregulated and caspase-3, 8, 9 and PARP cleaved. Data are representative of three individual experiments.

KAN0441571C induced a dose-dependent cytotoxic effect in all ROR1^+^ DLBCL cell lines while no effect could be noted in the ROR1 negative U2932 cell line (MTT assay). Cytotoxicity varied between the different cell lines. In comparison to venetoclax, KAN0441571C induced a similar or significantly better effect (Figure 2). Apoptosis was accompanied by downregulation of BCL-2, BCL-xL, and MCL-1 (Figure 3) and confirmed by Annexin V/PI staining (Figure S2) as well as by cleavage of PARP, caspases 3, 8, and 9 and upregulation of BAX protein in OCI-LY3 cell line (Figure 3). Furthermore, cell cycle arrest was induced as evident by the upregulation of the cell cycle control proteins p21, p27 and p53 (Figure S3).

It is well known that stromal cells promote tumor cell growth and increase the resistance to therapeutic agents [34]. The DLBCL cell line OCI-Ly3 was co-cultured with ROR1-negative stromal cells (HS-5) and KAN0441571C. A higher apoptotic rate of DLBCL cells in the absence of stromal cells was noted as compared to when stromal cells were present (Figure S4), indicating that the ROR1 inhibitor could only partially overcome the tumor-promoting effects of stromal cells in this system. Higher concentrations of the ROR1 inhibitor are needed to overcome the apoptotic inhibiting effect of stromal cells to achieve optimal tumor cell killing as also shown for CLL cells [29].

### 3.3. Effects on Signaling in DLBCL Cells

**Figure 4:**
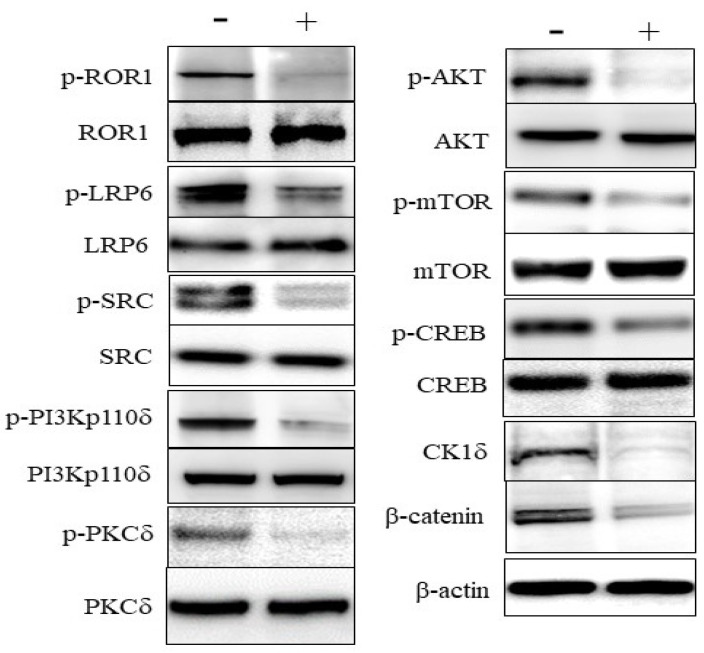
Effects of KAN0441571C (250 nM) (4 h) on ROR1 associated signaling molecules (Western blot) in the DLBCL cell line OCI-Ly3. ROR1, LRP6, SRC, PI3Kδ, PKC, AKT, mTOR, and CREB were dephosphorylated, CK1δ was downregulated and β-catenin was destabilized. Data are representative of three individual experiments.

KAN0441571C induced dephosphorylation of ROR1 as well as of the co-receptor LRP6 and the SRC protein which binds to phosphorylated ROR1. Furthermore, the downstream signaling molecules PKC and PI3Kδ/AKT/mTOR (non-canonical Wnt pathway), and Wnt/β canonical signaling molecule CK1δ was downregulated, β-catenin was destabilized as well as dephosphorylation of the transcription factor CREB (Figure 4). Venetoclax did not dephosphorylate ROR1 (Figure S5), nor did ibrutinib [29].

ROR1 and LRP6 were shown to be heterodimerized in OCI-Ly3. After 2–6 h of incubation with KAN0441571C, no dissociation of the ROR1/LRP6 complex could be seen (Figure S6), indicating that the major inhibiting effect of our SMI might not be by preventing binding of the ROR1 ligand (Wnt5a) to the receptor complex. Wnt5a is produced by tumor cells may act in an autocrine loop [35].

### 3.4. Effects of KAN0441571C in Zebra Fish Transplanted with DLBCL Cells

**Figure 5:**
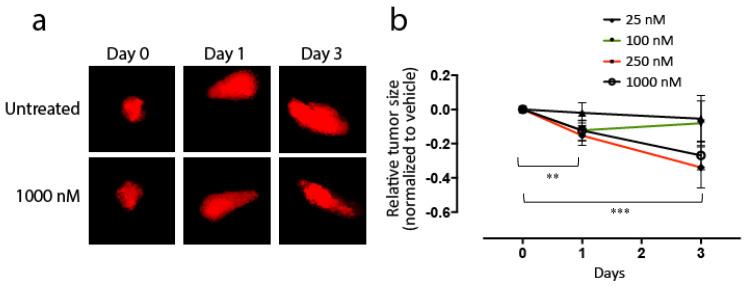
Zebra fish embryos were transplanted with the OCI-Ly3 cell line and treated with KAN0441571C for three days. (**a**) Photographs of only the tumor area days 0, 1 and 3 in zebra fishes transplanted with stained OCI-Ly3 in untreated and 1000 nM treated zebra fishes. (**b**) Tumor size (ratio) (mean ± SEM) in KAN0441571C treated zebra fishes at days 0, 1 and 3. A total of 21 zebra fish embryos were included in each treatment group. A statistically significant decrease in relative tumor size was noted in the treatment groups receiving the highest dose of KAN0441571C (250 nM and 1000 nM resp.) comparing day 0 with day 1 (** *p* < 0.01) and day 0 with day 3 (*** *p* < 0.001).

Zebra fish embryos transplanted with the OCI-Ly3 cell line were treated for three days with KAN0441571C (25–1000 nM). No toxic effects of the drug could be noted in the zebra fish. A 70% increase in the tumor area over the three-day period in untreated zebra fishes was seen. However, a significant dose- and time-dependent reduction in the tumor area was found in treated fishes (*p* = 0.005) (Figure 5a,b).

### 3.5. Effects of KAN0441571C in Combination with Venetoclax or Ibrutinib in ROR1^+^ DLBCL Cell Lines

**Figure 6:**
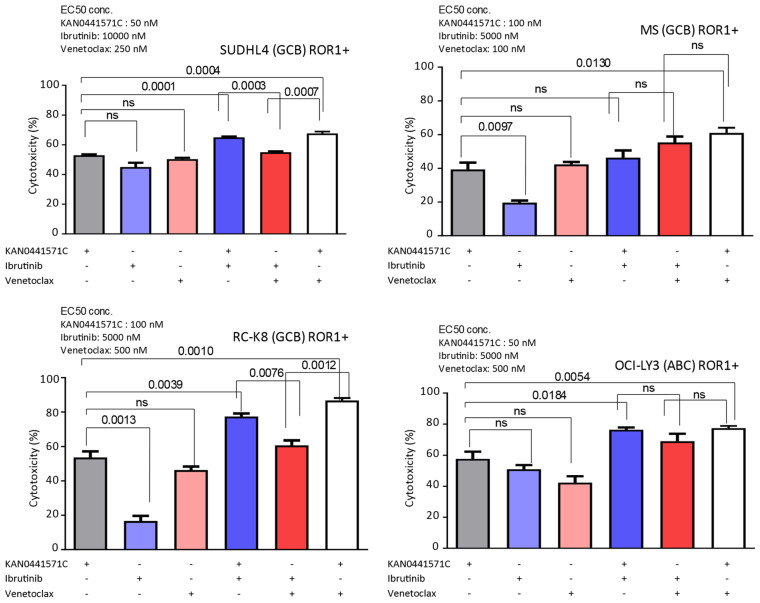
Cytotoxicity of four different ROR1^+^ DLBCL cell lines treated in vitro with combinations of EC_50_ concentrations of KAN0441571C, ibrutinib and venetoclax respectively (see upper left corner) alone and in combinations. Significant differences are shown at the top. Data present the mean ± SEM of three independent experiments. Using the Bonferroni method to correct for multiple testing all p-values below 0.002 remained significant while those above 0.0039 become non-significant

Several novel drugs are in the clinical development for DLBCL, including venetoclax and ibrutinib [36]. None of them dephosphorylated ROR1 in vitro (see above). EC_50_ for venetoclax in ROR1 positive DLBCL cell lines varied between 100 and 500 nM (Figure 2) and for ibrutinib between 5000 and 10.000 nM (data not shown). It should be noted that these small molecules have different mechanisms of action (MOA). EC_50_ concentrations of KAN0441571C for the respective cell line (Figure 2) were used to analyze cytotoxicity in combination with the EC_50_ concentrations for venetoclax and ibrutinib (Figure 6). KAN0441571C alone seemed to be superior to ibrutinib and similar efficacy as venetoclax. The combination of KAN0441571C and venetoclax resulted in nearly 100% killing of RC-K8 and OCI-LY3 tumor cells at the EC_50_ concentration of each drug (Figure 6).

## 4. Discussion

In the present study, we analyzed early clinical aspects of ROR1 expression in different DLBCL patient groups and performed in vitro and in vivo analyses of ROR1 inhibition of DLBCL cells. Our clinical observations were retrospective, made on small patient numbers and subsets and should thus be regarded as preliminary and hypothesis-generating only. The true value of ROR1 as a prognostic marker in DLBCL must be evaluated in prospective studies on large patient cohorts of newly diagnosed de novo DLBCL. Given these limitations, ROR1 expression in DLBCL was often observed in poor prognosis subgroups, such as primary refractory de novo DLBCL and Richter’s syndrome with the lowest frequency observed in non-relapsed DLBCL. It cannot be fully excluded that the high expression in Richter’s syndrome is, to some extent, biased by CLL. Among R/R de novo DLBCL patients, ROR1 positivity appeared to be associated with shorter overall survival, which in multivariable analysis was independent of some prognostic factors but not the frequently used clinical markers such as cell of origin and IPI. Notably, the prognostic value of IPI and cell of origin was outperformed by a recent prognostic model comprising genetic and functional drivers in DLBCL; in the very same study there was also a rather weak prognostic impact of ROR1 gene expression [37]. Our clinical observations, which were based on surface protein expression of ROR1, are in line with previous reports in various tumor types in which ROR1 was found to be associated with an inferior outcome [38]. In a recent report of DLBCL patients (*n* = 137) high ROR1 expression was significantly associated with B symptoms and Ann Arbor stage. The overall survival correlated significantly with ROR1 expression, B symptoms and LDH level, but only LDH was there an independent factor [25]. Collectively, data from most previous publications and the preliminary observations from the present study suggest that the expression of ROR1 may be related to poor prognosis and disease aggressiveness in DLBCL, a finding which is in line with reports in a variety of other malignancies.

To further address the significance of ROR1 in DLBCL, we performed functional studies on DLBCL cell lines, using a second generation small molecule ROR1 inhibitor, KAN0441571C, which was superior to our first generation [KAN0439834 [29]] in inducing apoptosis of DLBCL cells and with improved pharmacokinetics. KAN0441571C was similar or more effective than venetoclax in inducing apoptosis. When the two drugs were combined, additive apoptotic effects were observed, approaching almost complete tumor cell death. Thus, this combination might be of special interest to be explored further, as the drugs have different mechanisms of action. We also showed that KAN0441571C inhibited ROR1 phosphorylation in DLBCL cells and induced apoptosis is mainly through the intrinsic mitochondrial pathway, inhibiting pro-survival molecules (BCL-2 and MCL-1) and the upregulation of the pro-apoptotic BAX protein as well as cleavage of caspase 9 at low concentrations of the ROR1 inhibitor. However, caspase 8 was also cleaved at high concentrations of KAN0441571C, also supporting the activation of the extrinsic pathway. The drug inactivated both the Wnt canonical signaling pathway (CK1δ/β-catenin) and Wnt non-canonical pathway (PKC and PI3K/AKT/mTOR) as well as inactivated the transcription factor CREB. Finally, a zebra-fish model was applied for in vivo testing in which KAN0441571C induced a significant and consistent reduction of DLBCL cells.

In recent years, ROR1 has emerged as a molecule of substantial interest for the development of targeted therapy including mAbs [15], chimeric antigen receptor T-cells (CAR-T) [39,40], bi-specific T-cell engager (BiTE) [41] and SMIs targeting the cytoplasmic tyrosine kinase domain (TKI) [29,42] as well as the extracellular part of ROR1 [43,44]. The common characteristics for these compounds are that effects on signaling are similar.

In conclusion, the expression of the receptor tyrosine kinase ROR1 is associated with a poor prognosis in several malignancies including DLBCL and of importance for tumor cell proliferation survival, metabolism and epithelial-mesenchymal-transition (EMT). R/R DLBCL is still a major therapeutic challenge. There is a great medical need for new treatment alternatives for patients with refractory disease as well as those who have a relapse but are not candidates for high-dose chemotherapy with stem cell support. Targeting ROR1, which is involved in several signaling cascades of importance in tumors [10,44], might be a new rewarding approach. Various types of therapeutics against ROR1 are in the development, including the present SMI, targeting the intracellular TK domain of ROR1 developed by the phenotypic screening of CLL cells.

## 5. Conclusions

New agents are warranted in poor-prognosis DLBCL patients; the receptor tyrosine kinase ROR1 is absent in most normal adult tissues but overexpressed in several malignancies including DLBCL. In this study, we explored clinical and functional inhibitory aspects of ROR1 in DLBCL and showed—in preliminary analyses, and with a limited number of patients—that ROR1 expression was more often observed in primary refractory DLBCL than in relapsed and non-relapsed patients, that overall survival may be related to ROR1 expression, and that a second-generation small molecule ROR1 inhibitor induced the apoptosis of ROR1+ DLBCL cell lines in vitro and in a zebra fish model.

## Supplementary Materials

The following are available online at https://www.mdpi.com/2227-9059/8/6/170/s1, Table S1: Physicochemical differences comparing KAN0439834 and KAN0441571, Table S2: Factors on overall survival, Table S3: The ROR1 inhibitors KAN0439834 and KAN0441571C bind to a set of targets. The potencies (radiometric assay (ProQinase)) are lower than the binding affinities (displacement assay (DiscoverX)) on targets. Figure S1: Comparison of cytotoxicity of KAN0441571C and KAN0439834 on DLBCL cell lines, Figure S2: Apoptosis (Annexin-V/PI) of DLBCL cell lines after 24 h of incubation with KAN0441571C, Figure S3. Treatment or the DLBCL cell line OCI-LY3 with KAN0441571C (48 h) induced a dose-dependent growth inhibition and cell death (A, B) (cell counting and viability, trypan blue exclusion assay). Western blot analysis showed a dose-dependent increase in the levels of the cell cycle inhibitors p21 and p27 associated with an increase in the level of p53 (C, D), Figure S4: Apoptosis (Annexin V/PI) (24 h) in the DLBCL cell line, (OCI-Ly3) (ROR1^+^) co-cultured with HS-5 stromal cells (ROR1-) and KAN0441571C. OCI-Ly3 alone (10^5^ cells/well) (green line); 10^5^ OCI-Ly3 cells + 10^5^ HS-5 cells/well (red line); 10^5^ HS-5 cells/well (black line). Apoptosis of DLBCL cells were identified by gating for CD19, Figure S5: Effects on phosphorylation of ROR1 (pROR1) by venetoclax and KAN0441571C in OCI-LY3 cell line, incubated for 4 h, Figure S6. ROR1/LRP6 dimerization in OCI-Ly3 cell line. Representative staining images of untreated OCI-Ly3 cell line using anti-ROR1 and LRP6 antibodies in the in situ proximity ligation assay (PLA) (40X) and staining of OCI-Ly3 cells treated with KAN0441571C (2500 and 5000 nM, 6 h). Blue fluorescence (DAPI) was used for counterstaining. Pictures were captured by a fluorescent microscope (40×) (Scale bar: 20 µm).
