# Supplementary material for: ROR1 is Expressed in Diffuse Large B-Cell Lymphoma (DLBCL) and a Small Molecule Inhibitor of ROR1 (KAN0441571C) Induced Apoptosis of Lymphoma Cells
Source: Biomedicines. 2020 Jun 23;8(6):170. doi: 10.3390/biomedicines8060170 (PMC7344684; doi:10.3390/biomedicines8060170)
Supplement: Supplementary file 1 [file biomedicines-08-00170-s001.pdf]

Supplementary Materials

Table S1. Physicochemical differences comparing KAN 0439834 and KAN0441571.

Physicochemical characteristics	KAN0439834	KAN0441571
Molecular weight	535	555
$cLogD_{7.4}$ (calculated)	2.8	1.7
Polar surface area (Å^2) (calculated)	104	73

Table S2. Factors on overall survival.

Cox Proportional Hazard Models							
**Unadjusted**							
**Variable**	**n**	**events**	**censored**	**HR**	**CI-lower**	**CI-upper**	***p*-value**
ROR1 < 10%	33	25	8	0.43	0.19	0.96	**0.039**
**Adjusted for age**							
**Variable**	**n**	**events**	**censored**	**HR**	**CI-lower**	**CI-upper**	***p*-value**
				0.55	0.24	0.30	0.120
Age ≤ 60	33	25	8				
				0.50	0.16	1.51	0.218
**Adjusted for gender**							
**Variable**	**n**	**events**	**censored**	**HR**	**CI-lower**	**CI-upper**	***p*-value**
ROR1 < 10%				0.43	0.19	0.98	**0.046**
	33	25	8				
Gender: female				0.93	0.41	2.11	0.866
**Adjusted for clinical subtype**							
**Variable**	**n**	**events**	**censored**	**HR**	**CI-lower**	**CI-upper**	***p*-value**
ROR1 < 10%				0.34	0.14	0.82	**0.016**
	33	25	8				
Recurrent				1.77	0.72	4.37	0.215
**Adjusted for Ann Arbor stage**							
**Variable**	**n**	**events**	**censored**	**HR**	**CI-lower**	**CI-upper**	***p*-value**
ROR1 < 10%				0.43	0.19	0.96	**0.041**
	33	25	8				
Stage III or IV				0.84	0.36	1.96	0.685
**Adjusted for IPI**							
**Variable**	**n**	**events**	**censored**	**HR**	**CI-lower**	**CI-upper**	***p*-value**
ROR1 < 10%				0.52	0.23	1.20	0.127
	33	25	8				
IPI 1-2				0.37	0.16	0.85	**0.019**
**Adjusted for COO**							
**Variable**	**n**	**events**	**censored**	**HR**	**CI-lower**	**CI-upper**	***p*-value**
ROR1 <10%				0.54	0.22	1.32	0.18
	28	21	7				
COO (GC)				0.93	0.38	2.23	0.88

Table S3. The ROR1 inhibitors KAN0439834 and KAN0441571C bind to a set of targets. The potencies (radiometric assay (ProQinase)) are lower than the binding affinities (displacement assay (DiscoverX)) on targets.

Target	Mechanism	Affinity/Potency
ROR1	Apoptosis<br>EMT	< 25 nM IC50 (ROR1 dephosphorylation)
CDK 4/6/7/9	Cell cycle<br>Translation	Low nM binding/ low-high nM potency
Aurora A	Mitosis<br>EMT	Low-mid nM binding/ mid-high nM potency
FLT3	Leukemic blasts	Low nM Kd binding/ mid-high nM potency
JAK1,2,3/TYK2	Immune response	Low nM binding/ mid-high nM potency

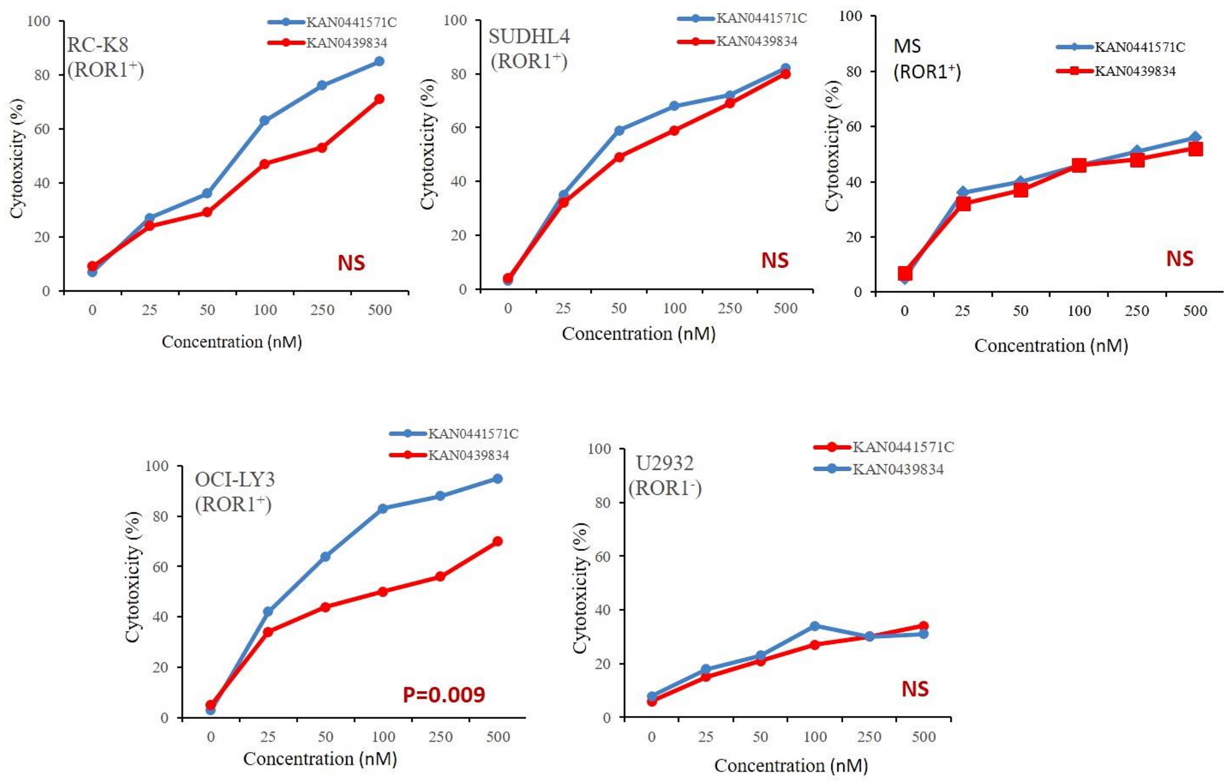

Figure S1. Comparison of cytotoxicity of KAN0441571C and KAN0439834 on DLBCL cell lines. For each concentration 6 replicates were used and each experiment was done twice.

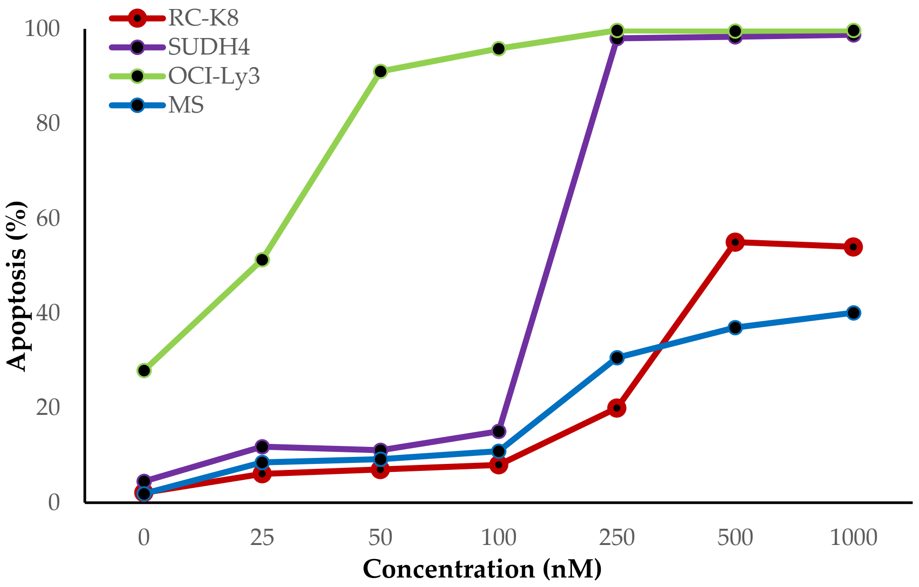

Figure S2. Apoptosis (Annexin-V/PI) of DLBCL cell lines after 24 h of incubation with KAN0441571C.

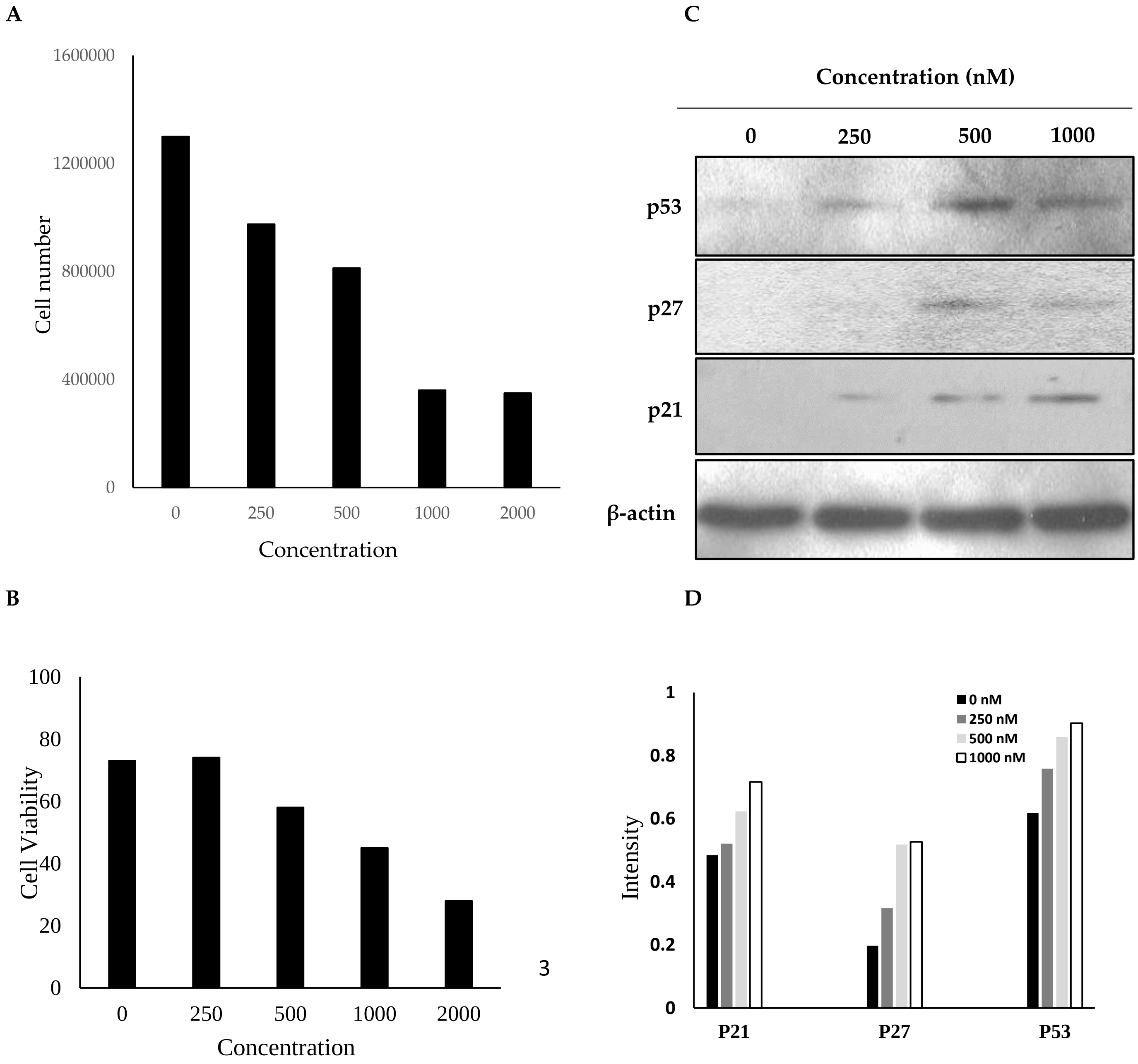

Figure S3. Treatment of the DLBCL cell line OCI-LY3 with KAN0441571C (48 h) induced a dose-dependent growth inhibition and cell death **(A, B)** (cell counting and viability, trypan blue exclusion assay). Western blot analysis showed a dose-dependent increase in the levels of the cell cycle inhibitors p21 and p27 associated with an increase in the level of p53 **(C, D)**.

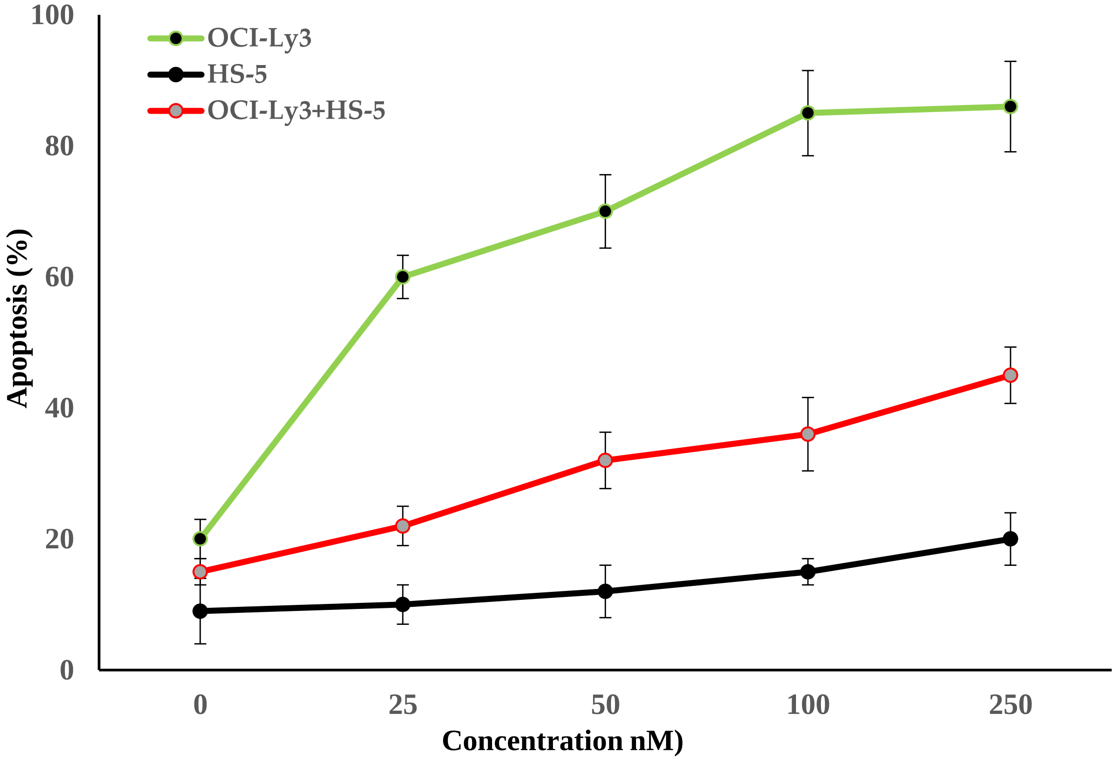

Figure S4. Apoptosis (Annexin V/PI) (24 h) in the DLBCL cell line, (OCI-Ly3) (ROR1+) co-cultured with HS-5 stromal cells (ROR1-) and KAN0441571C. OCI-Ly3 alone (10^5 cells/well) (green line); 10^5 OCI-Ly3 cells + 10^5 HS-5 cells/well (red line); 10^5 HS-5 cells/well (black line). Apoptosis of DLBCL cells were identified by gating for CD19. For each concentration experiment was done three times.

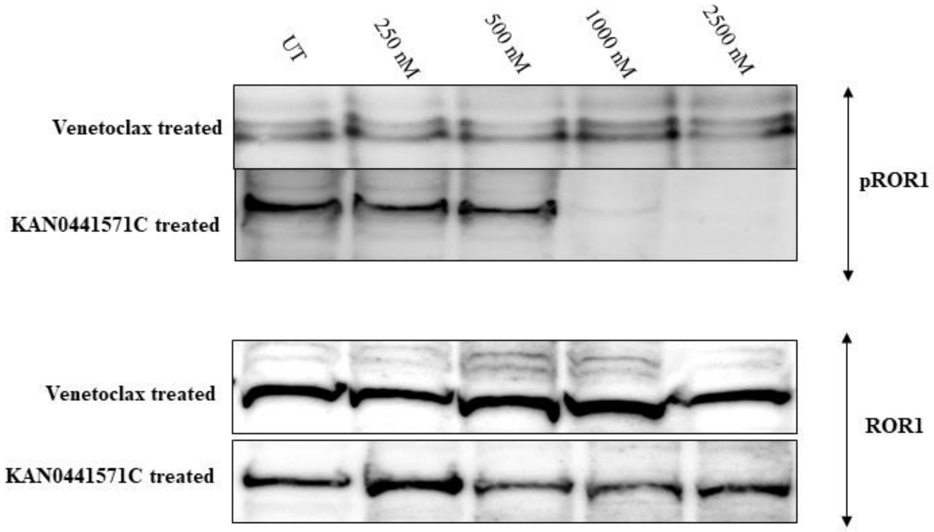

Figure S5. Effects on phosphorylation of ROR1 (pROR1) by venetoclax and KAN0441571C in OCI-LY3 cell line, incubated for 4 hours. Data are representative of three individual experiments.

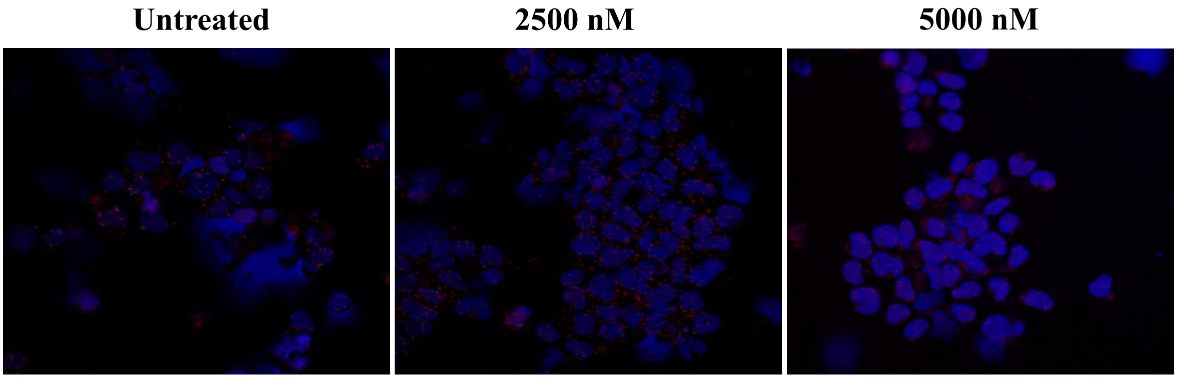

Figure S6. ROR1/LRP6 dimerization in OCI-Ly3 cell line. Representative staining images of untreated OCI-Ly3 cell line using anti-ROR1 and LRP6 antibodies in the in situ proximity ligation assay (PLA) (40×) and staining of OCI-Ly3 cells treated with KAN0441571C (2500 and 5000 nM, 6 h). Blue fluorescence (DAPI) was used for counterstaining. Pictures were captured by a fluorescent microscope (40×) (Scale bar: 20 μm).
